# The role of depression and use of alcohol and other drugs after partner suicide in the association between suicide bereavement and suicide: cohort study in the Danish population

**DOI:** 10.1017/S0033291724000448

**Published:** 2024-07

**Authors:** Alexandra Pitman, Keltie McDonald, Yanakan Logeswaran, Glyn Lewis, Julie Cerel, Gemma Lewis, Annette Erlangsen

**Affiliations:** 1 UCL Division of Psychiatry, 149 Tottenham Court Rd, London W1T 7AD, UK; 2 Camden and Islington NHS Foundation Trust, St Pancras Hospital, St Pancras Way, London NW1 0PE, UK; 3 Suicide Prevention & Exposure Lab, College of Social Work, University of Kentucky, Lexington, USA; 4 Danish Research Institute for Suicide Prevention – DRISP, Psychiatric Center Copenhagen, Copenhagen, Denmark; 5 Copenhagen Research Centre for Mental Health, Mental Health Center Copenhagen, Mental Health Services, Capital Region of Denmark, Copenhagen, Denmark; 6 Department of Mental Health, Johns Hopkins School of Public Health, Baltimore, USA; 7 Centre for Mental Health Research, The National Centre for Epidemiology and Population Health, ANU College of Health and Medicine, The Australian National University, Canberra, Australia

**Keywords:** depression, mediation analysis, substance-related disorders, suicide bereavement, suicide risk

## Abstract

**Background:**

Although suicide bereavement is associated with suicide and self-harm, evidence regarding mechanisms is lacking. We investigated whether depression and substance use (alcohol and/or other drugs) explain the association between partner suicide bereavement and suicide.

**Methods:**

Linkage of nationwide, longitudinal data from Denmark for the period 1980–2016 facilitated a comparison of 22 668 individuals exposed to bereavement by a partner's suicide with 913 402 individuals bereaved by a partner's death due to other causes. Using causal mediation models, we estimated the degree to which depression and substance use (considered separately) mediated the association between suicide bereavement and suicide.

**Results:**

Suicide-bereaved partners were found to have a higher risk of suicide (HR_adj_ = 1.59, 95% CI 1.36–1.86) and of depression (OR_adj_ 1.16, 95% CI 1.09–1.25) when compared to other-bereaved partners, but a lower risk of substance use (OR_adj_ 0.83; 95% CI 0.78–0.88). An increased risk of suicide was found among any bereaved individuals with a depression diagnosis recorded post-bereavement (OR_adj_ 3.92, 95% CI 3.55–4.34). Mediation analysis revealed that depression mediated 2% (1.68%; 95% CI 0.23%–3.14%; *p* = 0.024) of the association between suicide bereavement and suicide in partners when using bereaved controls.

**Conclusions:**

Depression is a partial mediator of the association between suicide bereavement and suicide. Efforts to prevent and optimize the treatment of depression in suicide-bereaved people could reduce their suicide risk. Our findings might be conservative because we did not include cases of depression diagnosed in primary care. Further work is needed to understand this and other mediators.

## Introduction

Consistent evidence demonstrates an association between suicide bereavement and subsequent risk of suicide (Pitman, Osborn, King, & Erlangsen, 2014, 2022). Apart from preventing exposure to suicide loss in significant others, intervention hinges upon identification of modifiable risk factors. However, we still lack insights into potential explanatory factors between suicide bereavement and suicide. These include psychiatric disorders (particularly depression), alcohol or other drug use, stigma, grief, loneliness, shared social and economic adversity, suicide suggestion, and genetic factors (inherited or due to homophily) (O'Connor & Nock, 2014; Pitman et al., 2014). It is important to understand these links to inform the content of post-suicide emotional support (postvention); an established policy adopted widely within suicide prevention strategies (Schlichthorst et al., 2022; World Health Organization, 2018). However, while evidence supports the effectiveness of postvention in reducing depression and anxiety there is no evidence it helps reduce risks of suicide (Andriessen et al., 2019; Linde, Treml, Steinig, Nagl, & Kersting, 2017; McDaid, Trowman, Golder, Hawton, & Sowden, 2008; Szumilas & Kutcher, 2011).

To date, the mechanisms underlying the elevated risk of suicide after suicide bereavement have not been investigated longitudinally to understand temporality. The two strongest candidate mediators are depression and substance (alcohol and/or other drugs) use, which are both prevalent after a suicide loss (Bolton et al., 2013; Erlangsen et al., 2017; Pitman et al., 2014; Spiwak et al., 2020) and each is associated with suicide (Fazel & Runeson, 2020). Qualitative studies in Australia, the UK, and the US provide accounts of depressive symptomatology after suicide bereavement (Entilli, Ross, de Leo, Cipolletta, & Kõlves, 2021; Pitman et al., 2018) linked to suicidality (Hunt, Young, & Hertlein, 2019), while longitudinal studies of suicide-bereaved partners and children confirm an increased probability of mood disorders (Appel et al., 2013; Erlangsen et al., 2017; Kuramoto et al., 2010; Pitman et al., 2014; Spiwak et al., 2020; Wilcox et al., 2010) and antidepressant treatment (Appel et al., 2016) after suicide loss. Qualitative research in the US and UK also documents excessive use of alcohol and other drugs after being bereaved by suicide (Eng et al., 2019; Hunt et al., 2019; Pitman, Stevenson, King, & Osborn, 2020) and other sudden unnatural causes (Drabwell et al., 2020; Pitman et al., 2020) as a means of coping with overwhelming thoughts and emotions. However, some individuals described reducing their use of alcohol or other drugs to better cope with emotions or to avoid substances perceived as contributing to the death of their relative or friend (Drabwell et al., 2020; Eng et al., 2019; Pitman et al., 2020). Longitudinal studies of suicide-bereaved offspring report an increased probability of alcohol or other drug use after suicide bereavement (Brent, Melhem, Donohoe, & Walker, 2009; Wilcox et al., 2010), possibly transient (Hamdan, Melhem, Porta, Song, & Brent, 2013), but longitudinal studies of suicide-bereaved partners find a similar prevalence of alcohol or other drug use disorders to partners bereaved by other causes (Bolton et al., 2013; Erlangsen et al., 2017; Spiwak et al., 2020). Such inconsistencies may be explained by differences in measurement and samples. It is also possible that mechanisms differ by kinship group (Pitman et al., 2022).

Given the evidence supporting depression and alcohol and/or other drug use as candidate mediators, large longitudinal studies are required to investigate this, ideally by comparing suicide bereavement to other bereavements to account for the experience of bereavement *per se*. New methods of counterfactual mediation analysis (MacKinnon, Fairchild, & Fritz, 2007) are underused in suicide research. These offer a more robust means of assessing the relative magnitude of different pathways and mechanisms by which an exposure may affect an outcome because it makes the causal assumptions explicit, adjusts for confounders of each arm of mediational models, and takes into account interactions with exposure (VanderWeele, 2016a). The aim of this study was to estimate the relative proportions of the association between suicide bereavement and suicide mediated by depression and by substance use (alcohol and/or other drugs) when comparing suicide-bereaved partners to other-bereaved partners.

## Methods

### Study design and participants

We conducted a population-based cohort study, analyzing data from administrative national registers linked using a unique personal identification number assigned to all individuals in Denmark (Erlangsen & Fedyszyn, 2015). Population data from the Civil Registration System was linked with information on psychiatric and somatic hospital contacts from the Psychiatric Central Research Register (since 1970) (Mors, Perto, & Mortensen, 2011) and the National Patient Register (since 1977) (Schmidt et al., 2015) but linkage was not available to primary care data.

We included all Danish-born individuals aged 16 years and older who were living in Denmark between 1 January 1980 and 31 December 2016, comparing participants bereaved by the (a) suicide or (b) non-suicide death of a current/former partner (spouses, civil partners, and cohabitees) over that period. Cohort inception in 1980 provided us with over 10 years of previous data on psychiatric confounders as identified in the Psychiatric Central Research Register.

We focused on studying mechanisms in one kinship group (partners) because they offered the largest sample relative to parents, offspring, and siblings (Pitman et al., 2022). Exposure to partner bereavement was identified by linking data on individuals in the cohort to deceased current and former spouses/cohabitees, identifying causes of death from the Register of Causes of Death. Partners (opposite and same-sex married and registered partners and opposite sex cohabitees) were identified using an established linkage method based on data recorded in the Civil Registration System (Pedersen, 2011) (online Supplemental Methods S1). Individuals entered the cohort on the date of the index (first) bereavement and were followed up until the date of their suicide, death from a cause other than suicide, migration out of Denmark, second partner bereavement, or the end of follow-up (31 December 2016).

### Measures

#### Outcome

Our outcome, suicide, was identified from the Register of Causes of Death (Helweg-Larsen, 2011) based on International Classification of Diseases (ICD)-8 and ICD-10 codes (online Supplemental Table S1).

#### Exposure

Relevant ICD-8 and ICD-10 codes were used to identify whether bereavement was due to suicide or other causes. The first partner bereavement over this period was identified as the index exposure, and individuals were censored at any subsequent partner bereavement.

#### Confounders

We chose eight confounders *a priori*, based on existing evidence (Pitman et al., 2014), which we used in each arm of the mediation model: sex; age; bereavement year; marital status (as distinct from cohabitation status, to capture the effect of divorce); household income level (quartiles); pre-bereavement history of any admission recording self-harm (in psychiatric or physical health settings); pre-bereavement psychiatric disorders recorded on any admission (including alcohol and/or other drug use); and pre-bereavement physical health conditions recorded on medical admission (online Supplemental Table S1). Bereavement year captured period effects and differing inception years for inpatient and outpatient data. All confounders were measured before the index bereavement.

#### Mediators

Depression was defined as a diagnosis recorded during psychiatric inpatient (since 1980) or outpatient (since 1995) contact. Substance use was defined broadly as an ICD-8/ICD-10 diagnosis of alcohol and/or other drug use disorders recorded during psychiatric inpatient (since 1980) or outpatient (since 1995) contact, including acute intoxication, as well as inpatient medical admission recording specific medical problems linked to alcohol use (Grissa, Rasmussen, Krag, Brunak, & Jensen, 2020). Both mediators were restricted to events recorded after the index bereavement.

### Statistical analysis

The demographic and clinical characteristics of the sample were examined using complete case analysis. We used Cox proportional hazards regression to examine the associations between suicide bereavement and time to suicide in unadjusted and adjusted models. We assessed model assumptions including proportionality of hazards (online Supplemental Methods S2). In preliminary analyses we accounted for potential clustering effects, where multiple current/former partners might be bereaved by the same death, using multilevel Cox proportional hazards regression clustered on the individual who died. As the multilevel model provided comparable estimates to the single-level model, we opted for the latter.

Mediation by depression and substance use was assessed separately using the causal inference potential outcomes framework to decompose the total effect (TE) of suicide bereavement on suicide into four components: (a) controlled direct effect (CDE), (b) reference interaction (INT_ref_), (c) mediated interaction (INT_med_); and (d) pure indirect effect (PIE), and other derived values, including the total indirect effect (TIE), portion attributable to interaction (PAI), and the portion eliminated (PE) (Richiardi, Bellocco, & Zugna, 2013; Vanderweele, 2014; VanderWeele, 2016b) (online Supplemental Methods S4; Supplemental Box S1). From this output, we identified: the overall proportion of the association mediated (equivalent to 100 × [TIE/TE]); the proportion of the association attributable to interaction (effectively 100 × [PAI/TE]); and the proportion of the total effect of the exposure on the outcome that would be eliminated in the absence of the mediator (effectively 100 × PE/TE). The last of these is of policy relevance in conveying how much of the effect of the exposure can be prevented by intervening on the mediator.

**Figure 1.**
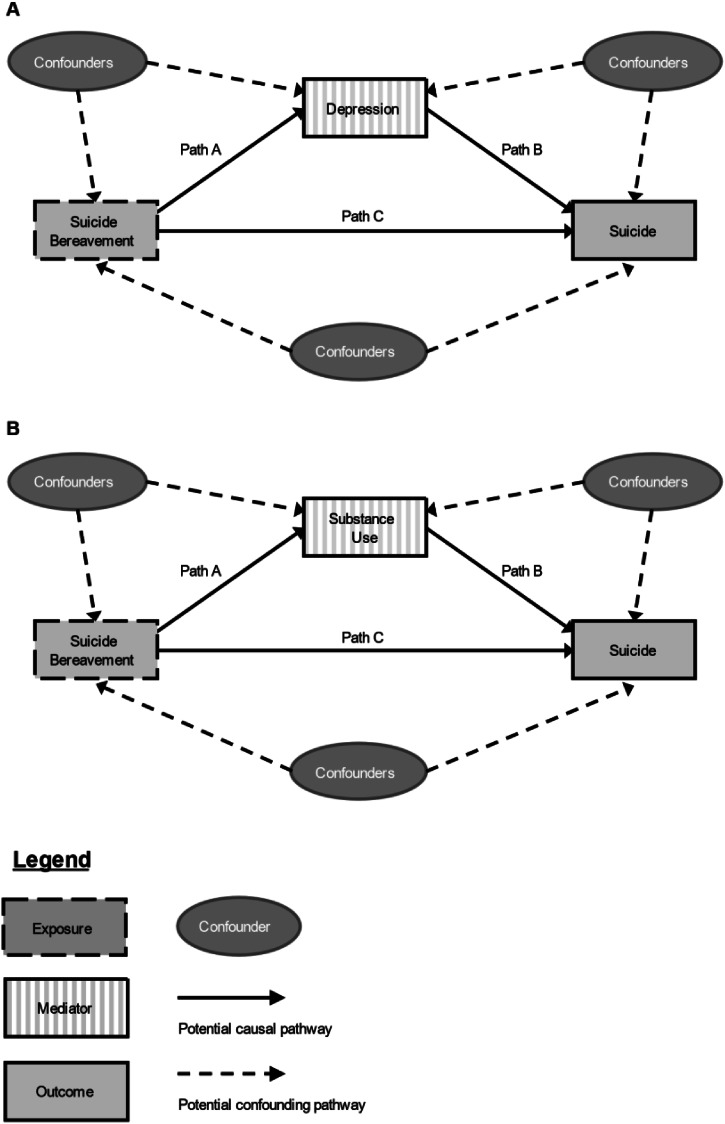
Hypothesized model for the mediating effect of (A) depression and (B) substance use on the association between suicide bereavement and suicide. Legend: Panel A displays the hypothetical model for mediation by depression. Panel B displays the hypothetical model for mediation by substance use. Solid lines represent potential causal pathways. Dashed lines reflect potential confounding pathways. Path A represents the association between suicide bereavement and post-bereavement depression/substance use (modeled using logistic regression). Path B represents the association between post-bereavement depression/substance use (in all those bereaved) and suicide modeled using logistic regression. Path C represents the association between suicide bereavement and suicide (modeled using Cox proportional hazards regression). The same set of confounders was used for all pathways: sex, age, bereavement year, legal marital status, household income level, pre-bereavement history of self-harm, any psychiatric disorders, and any physical disorders.

We fitted the mediation model using the *med4way* command in Stata (Discacciati, Bellavia, Lee, Mazumdar, & Valeri, 2019; StataCorp, 2019). The outcome model (path C in Fig. 1) was fitted using Cox proportional hazards regression, while the mediation model (paths A and B) was fitted using logistic regression because *med4way* does not accommodate a time-to-event mediator and time-to-event outcome.

In our first sensitivity analysis we assessed the influence of competing risks (death from other causes; second bereavement; emigration) in our main association (Supplemental Methods 3). We then conducted an interaction test to investigate whether partner status (ex- versus current) modified this main association. In our second sensitivity analysis we repeated our mediation analyses restricted to records of inpatient admissions to ascertain the influence of having incorporated outpatient contact data within our main analyses. In our third sensitivity analysis we repeated our mediation analyses excluding those with widowed marital status to ascertain the influence of having included those with additional partner bereavements prior to our period of interest.

We repeated our mediation analyses restricted to records of inpatient admissions to ascertain the influence of having incorporated outpatient contact data within our main analyses. We also repeated our mediation analyses excluding those with widowed marital status to ascertain the influence of having included those with additional partner bereavements prior to our period of interest.

Data management was performed using SAS software 9.4 (*SAS System for SunOP*, 2003) and analyses were performed using Stata 17 software (StataCorp, 2021).

## Results

### Sample characteristics

A total of 960 272 bereaved partners were identified of whom 24 202 (2.5%) were excluded due to missing data on income level (2.5%) and/or marital status (0.01%) (online Supplemental Table S2). Among the remaining 936 070 (97.5%) partners, 22 668 (2.4%) had been bereaved by suicide and 913 402 (97.6%) by other causes of death (Table 1). The majority of the analytic sample was female (67.3%). Individuals bereaved by a partner's suicide were more likely to be younger, female, to have a higher household income level and a past history of self-harm.

**Table 1.** Demographic and clinical features of the studied cohort (*n* = 936 070) according to bereavement status

	Suicide bereavement			Other bereavement		
Characteristic^a^	*N*	%	Person-years	*N*	%	Person-years
Total	22 668	2.4	342 523	913 402	97.6	9 342 742
Sex						
Male	6844	30.2	97 795	299 487	32.8	2 558 693
Female	15 824	69.8	244 727	613 915	67.2	6 784 031
Age (median, IQR)	Median	IQR		Median	IQR	
Bereavement (entry)	47	38–58	–	68	58–77	–
Exit	63	53–74	–	79	70–86	–
Bereavement year (median, IQR)	1995	1988–2005	–	1998	1990–2007	–
Status of deceased partner	*N*	%	Person-years	*N*	%	Person-years
Current partner	12 192	53.8	199 353	690 825	75.6	7 396 302
Ex-partner	10 465	46.2	142 969	222 377	24.4	1 944 338
Missing	11	<0.1	201	200	<0.1	2102
	Median	IQR		Median	IQR	
Time since split for ex-partners	6	2–13		9	3–18	
Household income level^b^	*N*	%	Person-years	*N*	%	Person-years
1 (lowest)	871	3.8	13 926	58 827	6.4	687 307
2	5573	24.6	69 613	507 523	55.6	4 552 310
3	7512	33.1	114 778	204 089	22.3	2 291 479
4 (highest)	8712	38.4	144 206	142 963	15.7	1 811 629
Marital status at bereavement^c^						
Never married	4054	17.9	61 617	48 177	5.3	505 863
Married/registered partnership	13 746	60.6	214 423	753 018	82.4	7 799 225
Divorced/dissolved partnership	4700	20.7	64 298	98 445	10.8	906 557
Widowed	168	0.7	2185	13 763	1.5	131 080
Pre-bereavement self-harm^d^	756	3.3	9174	15 133	1.7	122 360
Pre-bereavement psychiatric disorder^d^						
Any	2010	8.9	22 918	56 466	6.2	459 994
PTSD	9	<0.1	64	295	<0.1	1918
Depression	640	2.8	7416	21 556	2.4	183 810
Anxiety	246	1.1	3011	6317	0.7	55 024
Substance use	1264	5.6	13 056	31 287	3.4	233 141
SMI	471	2.1	5837	12 263	1.3	102 537
Pre-bereavement physical disorder^d^						
Any	1597	7.0	13 088	149 009	16.3	903 355
Cardiovascular disease	846	3.7	6771	94 106	10.3	556 706
Hypertension	354	1.6	2725	30 783	3.4	189 325
Diabetes mellitus	300	1.3	2800	22 094	2.4	132 434
COPD	261	1.2	1779	26 107	2.9	130 153
Post-bereavement measures						
Suicide deaths after exposure to bereavement	182	0.8	1270	3551	0.4	19 203
Depression after exposure to bereavement^e^	938	4.1	16 287	26 744	2.9	350 660
Substance use after exposure to bereavement^e^	1407	6.2	22 646	27 597	3.0	326 476

IQR, interquartile range; PTSD, post-traumatic stress disorder; COPD, chronic obstructive pulmonary disease; SMI, severe mental illness (defined as psychotic disorders, manic episode, bipolar affective disorder, and depression with psychotic symptoms).

a Data are *n* (%), except age, which is summarized as median (IQR). All values were pre-bereavement unless otherwise specified.

b Household income quartiles represent total income within the household divided by the total number of adults living in the household, then categorized into quartiles based on national annual income averages.

c The widowed category represented people who were bereaved by subsequent partner loss before the population registers had started, yet were bereaved while in a new partnership, so by default were registered as widowed by a former partner's death.

d Data for these variables were from diagnoses (or self-harm) recorded on inpatient admissions (i.e. excluding outpatient data).

e Data for these variables were from diagnoses recorded on inpatient admissions and outpatient contacts.

All *p*-values were <0.001 apart from depression (<0.01) and PTSD (non-significant) but these values are to be interpreted in the context of a large sample size, and therefore a high probability of statistically significant differences.

### Main association

During almost 10 million person-years of follow-up, 3733 individuals died by suicide, of whom 182 (4.9%) had been bereaved by partner's suicide and 3551 (95.1%) bereaved by other causes. Individuals bereaved by a partner's suicide had a higher risk of subsequent suicide compared to those bereaved by other causes (HR_adj_: 1.59, 95% CI 1.36–1.86) (Table 2). The absolute risk of suicide after partner bereavement was 0.80% in individuals bereaved by a partner's suicide and 0.39% in individuals bereaved by other causes.

**Table 2.** Hazard ratios and 95% confidence intervals for the association between suicide bereavement and suicide compared with other bereavement (Fig. 1, Path C)

	HR	95% CI	*p* value
Unadjusted	1.61	1.38–1.87	<0.001
Adjusted 1^a^	1.66	1.42–1.94	<0.001
Adjusted 2^b^	1.59	1.36–1.86	<0.001

HR, hazard ratio; CI, confidence interval.

a Adjusted for sex, age, bereavement year, legal marital status, and household income level.

b Final model: adjusted for all variables in adjustment 1, plus pre-bereavement history of self-harm, any psychiatric disorders (one binary variable), and any physical disorders (one binary variable).

### Mediation pathways

Suicide bereavement was associated with an increased risk of depression (Path A [exposure-mediator]; OR_adj_ 1.16, 95% CI 1.09–1.25). Post-bereavement depression was associated with an increased risk of suicide (Path B [mediator-outcome]: OR_adj_ 3.92, 95% CI 3.55–4.34; Table 3).

**Table 3.** Odds ratios and 95% confidence intervals for each pathway (modeled separately) in the model for mediation of the association between suicide bereavement and suicide by depression (Fig. 1, Panel A) and substance use (Fig. 1, Panel B)

			Unadjusted		Adjusted 1^a^		Adjusted 2^b^	
Model	Group	*N* (%) ^c^	OR	95% CI	OR	95% CI	OR	95% CI
Depression								
Path A (exposure-mediator)	Other-bereaved	26 744 (2.9%)	–	–	–	–	–	–
	Suicide-bereaved	938 (4.1%)	1.43*	1.34–1.53	1.14*	1.07–1.23	1.16*	1.09–1.25
Path B (mediator-outcome)	No depression	3223 (0.4%)	–	–	–	–	–	–
	Depression	510 (1.8%)	5.27*	4.80–5.80	5.50*	5.00–6.05	3.93*	3.55–4.34
Substance use								
Path A (exposure-mediator)	Other-bereaved	27 597 (3.0%)	–	–	–	–	–	–
	Suicide-bereaved	1407 (6.2%)	2.12*	2.01–2.25	0.81*	0.76–0.86	0.83*	0.78–0.88
Path B (mediator-outcome)	No substance use	3350 (0.4%)	–	–	–	–	–	–
	Substance use	383 (1.3%)	3.61*	3.25–4.02	2.77*	2.48–3.10	1.59*	1.41–1.79

OR, odds ratio; CI, confidence interval.

a Adjusted for sex, age, bereavement year, marital status, and household income level.

b Final model: adjusted for all variables in adjustment 1, plus pre-bereavement history of self-harm, any psychiatric disorders (one binary variable), and any physical disorders (one binary variable).

c *N* is solely for the outcome in each path; thus of those who are suicide-bereaved, 938 (4.1%) have depression and 1407 (6.2%) have substance use, and of those who are other-bereaved, 26744 (2.9%) have depression and 27597 (3.0%) have substance use. Of those who have depression, 510 (1.8%) die by suicide, and of those who do not have depression, 3223 (0.4%) die by suicide. Similarly, of those who have substance use, 383 (1.3%) die by suicide, and of those who do not have substance use, 3350 (0.4%) die by suicide.

* *p* < 0.001.

Suicide bereavement was associated with an increased risk of substance use in the unadjusted model (OR_crude_ 2.12; 95% CI 2.01–2.25; Table 3) but this attenuated and changed direction when adjusted (OR_adj_ 0.83; 95% CI 0.78–0.88). *Post hoc* adjustments (online Supplemental Table S3) showed that the attenuation was primarily due to the confounding effect of age, as the suicide-bereaved group was younger than controls, and older people had a lower probability of substance use. Nevertheless, post-bereavement substance use was associated with an increased risk of suicide (OR_crude_ 3.61; 95% CI 3.25–4.02; OR_adj_ 1.59, 95% CI 1.41–1.79).

### Mediation analyses

We estimated that the proportion of the association between suicide bereavement and suicide attributable to mediation by depression (100 × TIE/TE) was 1.68% (95% CI 0.23–3.14%; *p* = 0.024; Table 4), supporting depression as a partial mediator. We found no evidence to support an interaction between suicide bereavement and depression (100 × PAI/TE = 3.05%; 95% CI −4.47 to 10.59%; *p* = 0.427; online Supplemental Results S1). The proportion of suicide that would be prevented among the suicide-bereaved if intervening support could prevent depression (100 × PE/TE) was 4.35% (95% CI −3.29 to 12.00%; *p* = 0.264).

**Table 4.** Mediation analyses describing the role of depression in the association between suicide bereavement and suicide

		Adjusted estimate^a^		
Component	Stata output label^b^	RER	95% CI	*p* value
Total effect (TE)	*tereri*	0.57	0.32–0.82	<0.001
Total effect (TE) relative risk ratio (TE + 1)	*tereria*	1.57	1.32–1.82	<0.001
Decomposed into:				
(a) Controlled direct effect (CDE)	*ereri_cde*	0.55	0.30–0.80	<0.001
(b) Reference interaction (INT_ref_)	*ereri_intref*	0.02	−0.02 to 0.05	0.422
(c) Mediated interaction (INT_med_)	*ereri_intmed*	0.00	0.00–0.01	0.433
(d) Pure indirect effect (PIE)	*ereri_pie*	0.01	0.00–0.01	<0.001
Combinations:				
Total indirect effect (TIE)	computed	0.01	0.00–0.02	0.011
Portion attributable to interaction (PAI)	computed	0.02	−0.03 to 0.06	0.422
Portion eliminated (PE)	computed	0.02	−0.02 to 0.07	0.254
Proportions derived:				
% attributable to CDE	*p_cde*	95.65	88.00–103.29	<0.001
% Attributable to INT_ref_	*p_intref*	2.37	−3.91 to 9.25	0.426
% Attributable to INT_med_	*p_intmed*	0.38	−0.58 to 1.35	0.437
% attributable to PIE	*p_pie*	1.30	0.39–2.21	0.005
Overall % attributable to mediation by depression (equivalent to 100 × TIE/TE)	*op_m*	1.68	0.23–3.14	0.024
Overall % attributable to interaction (equivalent to 100 × PAI/TE)	*op_ati*	3.05	−4.47 to 10.58	0.427
% Eliminated (equivalent to 100 × PE/TE)	*op_e*	4.35	−3.29 to 12.00	0.264

RER, relative excess risk; CI, confidence interval; see online Supplemental Box S1 for definitions.

a Adjusted for sex, age, bereavement year, marital status, household income level, pre-bereavement history of self-harm, any psychiatric disorders (one binary variable), and any physical disorders (one binary variable). Note that in a mediation analysis, only adjusted estimates are presented because mediation can only be interpreted in the context of adjusting for all measured confounders (see online Supplemental Methods S4).

b Where no stata label is given, this value was computed from other stata output values.

In a separate model for substance use, we found no evidence to support a mediating role in the association between suicide bereavement and suicide (Table 5).

**Table 5.** Mediation analyses describing the role of substance use in the association between suicide bereavement and suicide

		Adjusted estimate^a^		
Component	Stata output label^b^	RER	95% CI	*p* value
Total effect (TE)	*tereri*	0.67	0.40–0.95	<0.001
Total effect (TE) relative risk ratio (TE + 1)	*tereria*	1.67	1.40–1.95	<0.001
Decomposed into:				
(a) Controlled direct effect (CDE)	*ereri_cde*	0.68	0.40–0.96	<0.001
(b) Reference interaction (INT_ref_)	*ereri_intref*	−0.01	−0.02 to 0.01	0.245
(c) Mediated interaction (INT_med_)	*ereri_intmed*	0.00	0.00–0.00	0.252
(d) Pure indirect effect (PIE)	*ereri_pie*	0.00	0.00–0.00	<0.001
Combinations:				
Total indirect effect (TIE)	computed	0.00	0.00–0.00	0.668
Portion attributable to interaction (PAI)	computed	−0.01	−0.02 to 0.00	0.245
Portion eliminated (PE)	computed	−0.01	−0.02 to 0.00	0.144
Proportions derived:				
% attributable to CDE	*p_cde*	101.25	99.67–102.83	<0.001
% attributable to INT_ref_	*p_intref*	−1.17	−3.06 to 0.71	0.221
% attributable to INT_med_	*p_intmed*	0.20	−0.12 to 0.52	0.229
% attributable to PIE	*p_pie*	−0.27	−0.42 to 0.11	0.001
Overall % attributable to mediation by depression (equivalent to 100 × TIE/TE)	*op_m*	−0.07	−0.41 to 0.26	0.678
Overall % attributable to interaction (equivalent to 100 × PAI/TE)	*op_ati*	−0.98	−2.55 to 0.59	0.222
% eliminated (equivalent to 100 × PE/TE)	*op_e*	−1.25	−2.83 to 0.33	0.122

RER, relative excess risk; CI, confidence interval; see online Supplemental Box S1 for definitions.

a Adjusted for sex, age, bereavement year, marital status, household income level, pre-bereavement history of self-harm, any psychiatric disorders (one binary variable), and any physical disorders (one binary variable). Note that in a mediation analysis, only adjusted estimates are presented because mediation can only be interpreted in the context of adjusting for all measured confounders (see online Supplemental Methods S4).

b Where no stata label is given, this value was computed from other stata output values.

### Sensitivity analyses

When accounting for competing risks in our main association (online Supplemental Table S4), similar results were obtained to those from the main analysis (crude sub-distribution hazard ratio [sHR]: 1.99, 95% CI 1.71–2.31; sHR_adj_: 1.54, 95% CI 1.32–1.80; online Supplemental Table S5). No interaction was found with respect to current/ex-partner status (*p* = 0.130; online Supplemental Table S6). When we restricted depression to inpatient records only for our main association, suicide bereavement was not associated with an increased risk of depression or substance use (online Supplemental Table S7), therefore we did not conduct mediation analyses using either measure. When we ran our mediation model for depression excluding those with widowed marital status, the proportion mediated rose from 1.68% to 1.74%, but with overlapping confidence intervals.

## Discussion

### Main findings

Based on complete, nationwide data on all bereaved partners, we found evidence of an elevated risk of depression and of suicide after partner suicide bereavement compared with bereaved controls, but a reduced risk of substance use. Suicide-bereaved partners had almost twice the risk of suicide when compared to partners bereaved by other causes. Bereaved partners with post-bereavement depression had almost a four-fold risk of suicide. Depression mediated at least 2% of the association between suicide bereavement and suicide, but for a number of reasons this could be an underestimate. Substance use was not found to mediate the association. Our definition of depression omitted any diagnoses made in primary care, where the majority of cases of depression in Denmark are treated (Musliner et al., 2019), cases diagnosed pre-1995 in outpatient care, and people never treated. Perceived stigma related to suicide might dissuade suicide-bereaved partners from seeking help (Hanschmidt, Lehnig, Riedel-Heller, & Kersting, 2016; Yang, Wong, Grivel, & Hasin, 2017), as could the perception that the system failed the deceased, resulting in under-ascertainment of psychiatric disorder and substance use when compared to controls. Finally, interventions offered during outpatient and inpatient care may also have mitigated suicide risk. For these reasons, it is plausible that depression mediates a greater proportion of the association between suicide bereavement and suicide than our study using secondary care data suggests. Further work is needed to investigate mediators of this association using both primary and secondary care data, as well self-report variables (where available) to capture perceived stigma and reluctance to seek help.

### Findings in the context of other studies

This is, to our knowledge, the first formal evaluation of potential mediators of suicide risk after suicide loss. Our findings extend existing evidence regarding the elevated risk of depression (Erlangsen et al., 2017) and suicide (Agerbo, 2003, 2005; Erlangsen et al., 2017) among suicide-bereaved partners compared with bereaved controls. In contrast, our novel finding of a reduced risk of substance use in the suicide-bereaved compared with bereaved controls (primarily due to the confounding effect of age) differs from previous findings of no differences (Bolton et al., 2013; Erlangsen et al., 2017). It is, however, consistent with the reduced risk of liver cirrhosis documented among suicide-bereaved spouses compared with bereaved controls (Erlangsen et al., 2017) and the accounts of people bereaved by suicide and other causes reporting a post-bereavement reduction in substance use (Drabwell et al., 2020; Eng et al., 2019; Pitman et al., 2020). This may be linked to the enhanced awareness described by suicide-bereaved individuals of their shared vulnerabilities to suicide, engendering a determination to safeguard their mental health after the loss (Pitman et al., 2017). We also noted that Danish partners bereaved by suicide had a significantly higher household income than partners bereaved by non-suicide causes, in keeping with a similar finding for bereaved partners in Canada (Spiwak et al., 2020) but in contrast with the converse finding for bereaved parents in Canada (Bolton et al., 2013).

### Strengths and limitations

Strengths include analyzing longitudinal data on a large population-based cohort of almost a million people, followed for approximately 10 million person-years, with minimal loss to follow-up, low levels of missing data, and avoidance of selection biases or recall biases. The Danish registers have been evaluated as reliable with respect to psychiatric hospital contacts (Tøllefsen et al., 2015) and registration of suicide death (Helweg-Larsen, 2011; Tøllefsen et al., 2015) and unnatural deaths (Tøllefsen et al., 2015), with good validity of diagnostic codes and the timing of admission/discharge dates in the Danish National Patient Register (Schmidt et al., 2015) and of depression diagnoses and admission/outpatient dates in the Psychiatric Central Research Register (Mors et al., 2011). For our study, this was important in separating out mediators and confounders for each pathway. In utilizing the Danish Civil Registration household variable, we were able to identify cohabiting couples as well as those in legal unions (although not opposite-sex cohabiting couples), providing a more realistic representation of Danish family structure and a more comprehensive measure of exposure to partner suicide loss. The limitations of using routine Danish registry data include the under-ascertainment of mental disorder and self-harm (due to lack of secondary care outpatient data pre-1995, primary care data, and data from people who do not seek or receive treatment) and not capturing other influences on suicide risk, such as personality traits, coping style, or social support.

Our application of contemporary causal inference methods overcame the limitations of older methods of mediation by allowing for the adjustment of confounders in each arm of the mediation model, making causal assumptions explicit, allowing us to model interactions, and was particularly suitable for binary outcomes(Rijnhart, Valente, MacKinnon, Twisk, & Heymans, 2021). However, mediation assumes the absence of residual confounding between exposure-outcome, exposure-mediator, or mediator-outcome, and we acknowledge the possibility of residual confounding due to unmeasured pre-bereavement depression or self-harm (not captured in settings beyond psychiatric admission). Our mediation models could not assess the role of the other respective putative mediator as an intermediate confounder because *med4way* can only consider one mediator at a time, for ease of interpretability. Our use of this model also relied on a set of theoretical assumptions, and it is possible that not all were satisfied, introducing the potential for biased estimates of causal effects.

We also acknowledge the potential for collider bias where selecting bereaved individuals for comparison might result in distorted associations between variables (Holmberg & Andersen, 2022). Given the difficulties in aligning follow-up for a cohort comprising individuals bereaved in childhood, early adulthood, mid-life, and late life, and because mechanisms may differ by kinship, we opted only to assess bereaved partners. This diminished statistical power to detect mediation effects. As a putative mediator, our broad substance use definition (including medical complications) relied less on a clinician recording an alcohol and/or other drug use disorder diagnosis e during admission or on treatment-seeking for perceived substance use problems. However, our narrow definition of depression omitted untreated depression, pre-1995 cases of depression in outpatients, cases identified in primary care (as we lacked primary care) or during somatic admissions, or information from prescribing data (available from 1995). Ascertainment bias is also possible if suicide-bereaved individuals are more likely to be in contact with mental health professionals and therefore more likely to be admitted.

### Clinical, policy, and research implications

The increased risk of depression after suicide loss and its contribution to suicide risk suggest a benefit of early identification of depression in suicide-bereaved partners (both ex- and current). Suicide risk mitigation might be achieved through appropriate bereavement support (Andriessen et al., 2019; Linde et al., 2017; McDaid et al., 2008; Szumilas & Kutcher, 2011), improving uptake through collaboration between bereavement services and primary care. Future research should use primary care and prescribing data to capture psychiatric disorders treated in primary and secondary care, investigating depression, substance use, and other putative mediators to advance our understanding of therapeutic targets. This will inform recommendations regarding appropriate support after suicide loss. The inclusion of postvention within a national suicide prevention strategy is not associated with changes in suicide mortality(Schlichthorst et al., 2022). However, a better understanding of mediators has the potential to improve the effectiveness of postvention and reduce suicides.

## Conclusions

Suicide risk in suicide-bereaved partners was almost twice that for partners bereaved by other causes. Depression mediated a small proportion (2%) of this association, but this study likely under-estimated the mediating role of depression by omitting depression treated in primary care. Risk of substance use was reduced after partner suicide loss, and substance use did not mediate the association between suicide bereavement and suicide. These findings, from a large, longitudinal representative dataset, identified early treatment of depression as a potential means of reducing the burden of suicide after suicide bereavement.

## Supporting information

Pitman et al. supplementary material Pitman et al. supplementary material

## Supplementary material

The supplementary material for this article can be found at https://doi.org/10.1017/S0033291724000448
