# Supplementary material for: The role of depression and use of alcohol and other drugs after partner suicide in the association between suicide bereavement and suicide: cohort study in the Danish population
Source: Psychol Med. 2024 Mar 11;54(9):2273–82. doi: 10.1017/S0033291724000448 (PMC11413345; doi:10.1017/S0033291724000448)
Supplement: Pitman et al. supplementary material [file S0033291724000448sup001.docx]

The role of depression and alcohol or drug use after partner suicide in the association between suicide bereavement and suicide: cohort study in the Danish population

Supplemental Materials

5 October 2023

Contents

Supplemental Methods 1: Explanation of the Danish registers’ definition of a partner (whether married or cohabiting) 2

Supplemental Methods 2: Assessment of proportional hazards assumption 3

Supplemental Methods 3: Sensitivity analyses to examine the possible influence of competing risks 3

Supplemental Methods 4: Mediation by depression and substance use 3

Supplemental Table 1. Derivation of key measures based on data sources and diagnostic classifications 5

Supplemental Box 1: Definitions of components or combinations of the total effect produced by four-way decomposition, with applications to the current study 8

Supplemental Figure 1. Log-log plot to assess proportionality of hazards 12

Supplemental Table 2. Demographic and clinical features of the analytic sample with complete information and the excluded sample with missing information 13

Any figures quoted as <3 indicate that cell size was below the threshold for reporting exact figures, as per the Statistics Denmark stipulations on protecting confidentiality. 14

Supplemental Table 3. Stepwise adjustments in the associations between a) suicide bereavement and depression, and b) suicide bereavement and substance use 15

Supplemental Table 4. Reason for cohort exit by bereavement status (representing competing risks) 15

Supplemental Results 1: 16

Supplemental Table 5: Sensitivity analysis to assess the association between suicide bereavement and suicide risk, accounting for competing risks 16

Supplemental Table 6. Hazard ratios and 95% confidence intervals for the association between suicide bereavement and suicide, stratified by partner status 16

Supplemental Table 7. Sensitivity analysis to assess the influence of using inpatient admission data only on each pathway of the mediation models 17

Supplemental Table 8. Proportions of sample with multiple bereavements 18

Supplemental Table 9. Sensitivity analysis for the depression mediation model, excluding those with widowed marital status 19

Supplemental References 20

# Supplemental Methods 1: Explanation of the Danish registers’ definition of a partner (whether married or cohabiting)

Statistics Denmark have developed an established linkage method for identifying partners (married or cohabiting) in the Danish Civil Register(Danmarks Statistik, 2022). This is based on family type, personal identification number, partner’s personal identification number, and household identification number, as recorded (since 1968) in the Civil Registration System (CRS). The personal identification numbers are known as CRS numbers.

A legally married/registered couple are defined as:

- Any married couple (different or same sex; joined through civil marriage or religious marriage) with or without children under 18 years living at home.

A cohabiting couple are defined as:

- Any cohabiting couple (identified through a record of joint children, via linkage of children to parents’ respective identification numbers), with or without children under 18 years living at home.
- Any cohabiting couple (different sex; age difference under 15 years, no indication of being genetically related, only two adults in the household aged 16+ years), with or without children under 18 years living at home.

Note that this definition of cohabitees excludes same-sex cohabiting couples, but any definition that included same sex cohabiting couples would over-estimate this number due to the high likelihood of misclassification of same-sex roommates as couples.

Note that these definitions may also include any child under 18 living with a person who has been married to one of their biological parents. This is conditional on that step-parent not having remarried, in which case the connection can no longer be made between the child and its stepfather or stepmother.

For our study we defined a partner as a cohabitee or legal spouse, as per the above.

# Supplemental Methods 2: Assessment of proportional hazards assumption

We assessed our models for violation of the proportional hazards assumption using log-log plots and a Schoenfeld residuals test, as well as visual inspection of plots of Schoenfeld residuals. A Schoenfeld residuals test based on adjusted model 2, was significant suggesting evidence against proportional hazards (global test: X^2^(12)=106.1.8, p<0.001). However, visual inspection of scaled Schoenfeld residuals for each term in the model (not shown) and a log-log plot suggested little departure from proportionality (**Supplemental Figure 1**).

# Supplemental Methods 3: Sensitivity analyses to examine the possible influence of competing risks

To assess the robustness of our results for the main association when taking into account the possible influence of i) other deaths, ii) experiencing a second bereavement or iii) emigration as competing risks for suicide, we fitted Fine and Gray (Fine & Gray, 1999) competing risks regression models. We fitted unadjusted and adjusted models for the effect of suicide bereavement on risk of suicide, after accounting for death from other causes, experiencing a second bereavement, and emigration as potential competing risks.

# **Supplemental Methods 4:** Mediation by depression and substance use

We used logistic regression to determine whether suicide bereavement was associated with depression (**Path A; Figure 1, Panel A**) and whether depression was associated with suicide (**Path B; Figure 1, Panel A**). The association between suicide bereavement and time to suicide was assessed using Cox proportional hazards regression (**Path C; Figure 1, Panel A**). For each path, we fitted unadjusted and adjusted models. We took the same approach for substance use as putative mediator (**Panel B**). We assessed mediation by depression (**Figure 1, Panel A**) and by substance use (**Figure 1, Panel B**), using a ‘potential outcomes’ framework (see below).

We performed four-way decomposition of mediating and interacting effects using the *med4way* command in Stata (Discacciati et al., 2019). Using a potential outcomes or counterfactual framework, it is possible to estimate the overall effect of an exposure on an outcome, in the presence of potential mediation and exposure-mediator interaction (Vanderweele, 2014a).

The total effect [TE] of exposure on outcome can be represented by: TE = CDE + INT_ref_ + INT_med_ + PI (**Supplemental Box 1**), where TE is the sum of the (a) controlled direct effect [CDE], the portion of effect due to neither mediation nor interaction (fixing the mediator to absent), (b) reference interaction [INT_ref_], the portion of effect due to interaction alone (the additive interactive effect when the mediator is left to what it would have been in the absence of exposure); (c) mediated interaction [INT_med_], the portion of effect due to both mediation and interaction; and (d) pure indirect effect [PIE], the portion of effect due to mediation alone.

As with other causal mediation analysis methods, the interpretation of causal effects requires four strong assumptions:

1. No unmeasured confounding of the exposure-outcome association
2. No unmeasured confounding of the mediator-outcome association
3. No unmeasured confounding of the exposure-mediator association
4. No confounder of the mediator outcome association is caused by exposure.

The use of counterfactuals also requires a strong assumption of sequential ignorability, which states that (a) conditional on all confounders, the exposure is independent of all potential values of outcome and mediator and (b) all potential outcomes are independent of the mediator given the observed exposure and confounders (Imai, Keele, & Tingley, 2010; Imai, Keele, & Yamamoto, 2010). When these assumptions are satisfied, we can produce unbiased estimates of causal effects. The potential outcomes framework can accommodate time-to-event outcomes and valid effect decomposition can be obtained using proportional hazards when the outcome is rare (VanderWeele, 2011).

In this study, we were interested in the mediating and interacting effects of depression and substance use. We aimed to assess mediators one at a time, as the use of multiple mediators within causal mediation frameworks is complex and hard to interpret. Notably, when mediators are considered one at a time, the sum of proportions mediated may be more than 100% due to mediators influencing one another (i.e. mediator-mediator interaction) (Vanderweele & Vansteelandt, 2013).

We interpreted the findings of this model in the context of the following:

- Whilst theoretical mediation models correspond to unobservable relations among variables, empirical mediation models correspond to statistical analyses of available data, inferring the true state of mediation from observations.
- Risk of depression and substance use might be influenced by a different set of factors at each of these points in the life course, complicating confounder selection.

Supplemental Table 1. Derivation of key measures based on data sources and diagnostic classifications

| **Measures** | **ICD-8 (1969-1993) code** | **ICD-10 (1994-2016) code** | **Population register** |
| --- | --- | --- | --- |
| **Causes of death** |  |  | Register of Causes of Death [since 1970] |
| Suicide | E950-E959 or where manner of death was recorded as 'suicide'. | X60-X84 and Y87.0 or where manner of death was recorded as 'suicide'. |  |
| Other cause of death | All codes for cause of death as recorded in the Causes of Death Register but excluding suicide (defined as above) | All codes for cause of death as recorded in the Causes of Death Register but excluding suicide (defined as above) |  |
| **Socio-demographic variables** |  |  |  |
| Birth sex (male/female) ^a^ | n/a | n/a | Civil Registration System [since 1968] |
| Age ^a^ | n/a | n/a | Civil Registration System [since 1968] |
| Marital status^a, b^ (never married; married; widowed; divorced/separated) | n/a | n/a | Civil Registration System [since 1968] |
| Household income level ^a,b,c^ | n/a | n/a | Registry of Social Pension and Income [since 1980] |
| **Self-harm**^a , d^ | E950-E959 or where ‘reason for contact’ was recorded as ‘self-harm’ | X60-X84 or when data on 'reason for contact' was recorded as ‘self-harm’ | Psychiatric Central Research Register [since 1969];  National Patient Register [since 1977] **^e^** |
| **Psychiatric disorders** ^a^ **^,^** ^f^ |  |  | Psychiatric Central Research Register [since 1969] |
| Depression | 296.09, 296.2, 298.0, 300.4 | F32.0-F32.2, F32.8-F32.9, F33.0-F33.2, F33.4-F33.9 |  |
| Anxiety | 300.0, 300.2 | F40, F41 |  |
| PTSD | 309.81 | 43.1 |  |
| Mental and behavioural disorders due to use of alcohol | 291, 303 | F10 (excluding F10.0) |  |
| Mental and behavioural disorders due to use of drugs | 304 | F11-F19 (excluding F1x.0) |  |
| SMI | 295, 296.89, 298.29-298.99, 299, 301.83, 296.1, 296.3, 298.1, 296.0 (excluding 296.09) | F20-F29, F30-F31, F32.3, F33.3 |  |
| **Physical Disorders** ^a^ ^, f^ |  |  | National Patient Register [since 1977] **^e^** |
| Cardiovascular disease | 413, 410, 420, 425, 427.91, 427.93, 427.94, 427.09, 427.10, 427.11, 427.19, 427.99 | I20-I22, I30, I42, I44, I46.0, I47.2, I48, I50 |  |
| COPD | 491-492, 518 | J41-J44, J47 |  |
| Diabetes Mellitus | 250 | E105, E109, E111, E115, E119, E131, E135, E139, E141, E145, E149 |  |
| Hypertension | 400-404 | I10-I13, I15 |  |
| **Variables used as putative mediators^g^** |  |  |  |
| Depression | 296.09, 296.2, 298.0, 300.4 | F32.0-F32.2, F32.8-F32.9, F33.0-F33.2, F33.4-F33.9 | Psychiatric Central Research Register [since 1969, with outpatient data included from 1995] |
| Substance use |  |  |  |
| Mental and behavioural disorders due to use of alcohol | 291, 303 | F10 (including F10.0) | Psychiatric Central Research Register [since 1969, with outpatient data included from 1995] |
| Mental and behavioural disorders due to use of drugs | 304 | F11-F19 (including F1x.0) | Psychiatric Central Research Register [since 1969, with outpatient data included from 1995] |
| Specific physical health complications of alcohol use | liver cirrhosis 571 | alcoholic fatty liver DK700;  alcoholic hepatitis DK701;  alcoholic fibrosis and sclerosis of liver DK702;  alcoholic cirrhosis of liver DK703;  alcoholic hepatic failure DK704;  alcoholic liver disease, unspecified DK709;  ascites R18;  oesophageal varices I185 | National Hospital Register [since 1977] |

**Legend**: ICD-8: International Classification of Disease 8^th^ revision; ICD-10: International Classification of Disease 10^th^ Revision. Note that ICD-9 was never implemented in Denmark, hence the transition from ICD-8 to ICD-10.

PTSD: post-traumatic stress disorder; COPD: chronic pulmonary obstructive disease; SMI: severe mental illness (defined as psychotic disorders, manic episode, bipolar affective disorder, and depression with psychotic symptoms)

^a^ Covariates used in final models, all measured prior to index bereavement.

^b^ Information in the Danish registers on marital status and income are updated on 1 January each calendar year, while all other covariates are updated on the date of a change (e.g. date of a physical health diagnosis being made in hospital). For marital status and income, we identified the last non-missing values prior to the index bereavement. Data on self-harm, psychiatric and physical health diagnoses covariates were identified as the recording of any of these during a hospitalisation prior to the index bereavement. Note that legal marital status was used as a confounder instead of cohabitation status to capture the confounding effect of divorce (Kposowa, 2000).

^c^ Household income level was calculated as the total household income (Baadsgaard & Quitzau, 2011), divided by the total number of adults within the household and categorised into quartiles based on the national annual income averages for that year in Denmark.

^d^ Self-harm was identified from relevant ICD-8 and ICD-10 codes, or where the reason for contact was recorded as self-harm.

^e^ The National Patient Register is sometimes termed the National Hospital Register.

**^f^** Applies to codes/diagnoses recorded on inpatient admissions. Psychiatric disorders and physical disorders were each added to models as separate binary variables.

**^g^** Applies to codes/diagnoses recorded on inpatient admissions and outpatient contact. All were measured after bereavement.

# Supplemental Box 1: Definitions of components or combinations of the total effect produced by four-way decomposition, with applications to the current study

| **Components** ^a^ | **Definition** ^b^ | **Application to depression in our model** ^c^ |
| --- | --- | --- |
| Total effect (TE)  TE = CDE + INT_ref_ + INT_med_ + PIE  (Y_11_ – Y_00_)  [*tereri*]  [note that the output term *tereria* provides TE + 1 ie as a Relative Risk Ratio] | The total effect of exposure on outcome. | The risk of suicide among individuals bereaved by suicide relative to individuals bereaved by other causes. |
| Controlled direct effect (CDE)  (Y_10_ – Y_00_)  [*ereri_cde*]  [note that the output term *p_cde* presents the proportion attributable to CDE] | The effect of the exposure on outcome that is due neither to mediation nor interaction (i.e., the direct effect of the exposure unexplained by the mediator). | The risk of suicide among individuals bereaved by suicide relative to individuals bereaved by other causes if no-one had depression (which could be understood statistically as a situation where depression was removed i.e. fixed to 0, or where risk operated through pathways that do not involve depression).  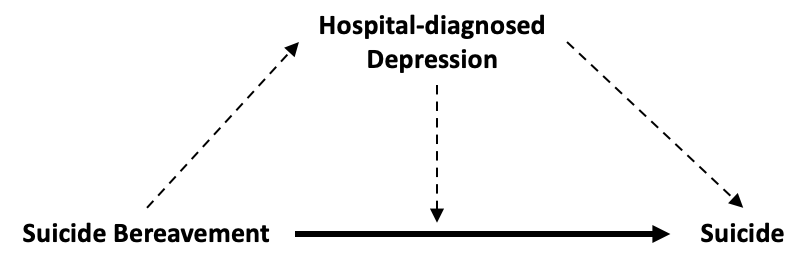 |
| Reference interaction (INT_ref_)  (Y_11_ – Y_10_ – Y_01_ + Y_00_)*(M_0_)  [*ereri_intref*]  [note that the output term *p_intref* presents the proportion attributable to INT_ref_] | The effect of exposure on outcome that is due to interaction but not mediation (i.e., the effect when the exposure operates in the presence of the mediator with the exposure itself not necessary for the mediator to be present). | The risk of suicide among individuals who are suicide-bereaved that operates when suicide bereavement is not a pre-requisite for depression.  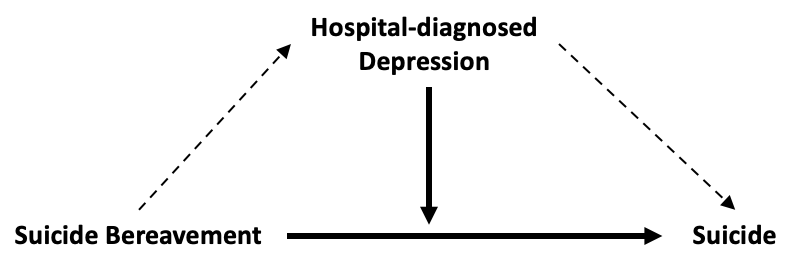 |
| Mediated interaction (INT_med_)  (Y_11_ – Y_10_ – Y_01_ + Y_00_)*(M_1_ – M_0_)  [*ereri_intmed*]  [note that the output term *p_intmed* presents the proportion attributable to INT_med_] | The effect of exposure on outcome that is due to mediated interaction (i.e., both interaction and mediation). This is the effect when the exposure operates in the presence of the mediator, with the exposure itself necessary for the mediator to be present (i.e. exposure causes the mediator, and the presence of the mediator is necessary for the exposure to have an effect on the outcome). | The risk of suicide among individuals who are suicide-bereaved that operates when suicide bereavement is a pre-requisite for depression.  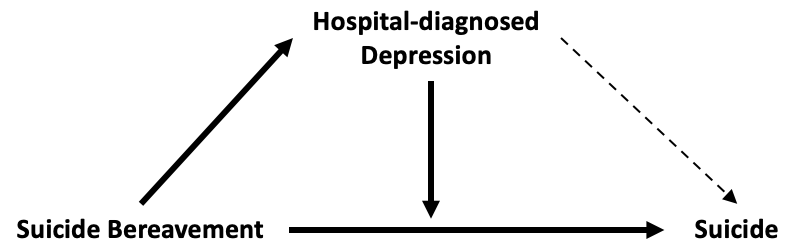 |
| Pure indirect effect (PIE)  (also known as the mediated main effect)  (Y_01_ – Y_00_)*(M_1_ – M_0_)  [*ereri_pie*]  [note that the output term *p_pie* presents the proportion attributable to PIE] | The effect of the exposure on outcome that is due to mediation alone (i.e., the indirect effect of the exposure that acts through the mediator). This is the effect when the mediator causes the outcome in the absence of the exposure and the exposure has an effect on the mediator itself. | The risk of suicide amongst those with depression, if suicide bereavement is a pre-requisite for depression.  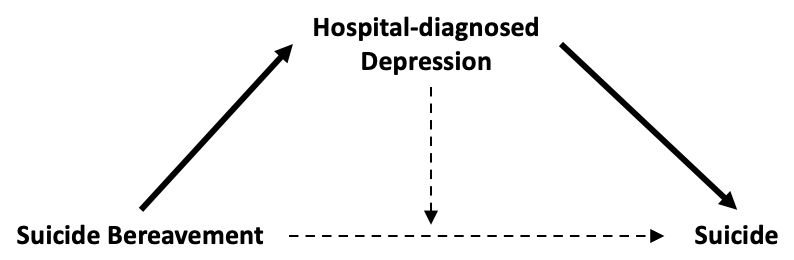 |
| Total Indirect Effect (TIE):  PIE + INT_med_  This is the portion attributable to mediation when expressed as a proportion: 100*TIE/TE)  [*op_m*] | The effect of the exposure on the outcome that is due to mediation (often expressed as a proportion of the total effect). | The effect of suicide bereavement on suicide that is due to mediation by depression. |
| Pure Direct Effect (PDE):  CDE + INT_ref_  [not provided in Stata output, but computed from this using the *nlcom* command] | The effect of the exposure on the outcome when the presence of the mediator is not necessary for the exposure to have an effect on the outcome. | The effect of suicide bereavement on suicide that is not mediated by depression. |
| Total Direct Effect (TDE):  CDE + INT_ref_ + INT_med_  [not provided in Stata output, but computed from this using the *nlcom* command] | The effect of the exposure on the outcome that does not act directly through the mediator (i.e. the direct effect of exposure on outcome, and the indirect effect of exposure on outcome through interaction with the mediator). | The effect of suicide bereavement on suicide that does not act directly through depression (i.e. the direct effect of suicide bereavement on suicide, and the indirect effect of suicide bereavement on suicide through interaction with depression). |
| Portion attributable to interaction (PAI):  INT_ref_ + INT_med_  (where expressed as a proportion: 100*PAI/TE)  [*op_ati*] | The effect of the exposure on the outcome that is due to interaction (often expressed as a proportion of the total effect). This is the effect when the exposure operates in the presence of the mediator, regardless of whether or not the exposure itself is necessary for the mediator to be present. | The effect of suicide bereavement on suicide that is due to interaction between depression and suicide bereavement. |
| Portion eliminated (PE):  INT_ref_ + INT_rned_ + PIE  (where expressed as a proportion:  100*[ INT_ref_ + INT_rned_ + PIE]/TE)  [*op_e*] | The effect of the exposure on the outcome that would be eliminated in the absence of the mediator (often expressed as a proportion of the total effect). | The effect of suicide bereavement on suicide that is due to depression either through mediation or interaction. This value is of interest for policy reasons as it relates to the proportion of cases of suicide that would be prevented among the suicide-bereaved if services intervened early so that no suicide-bereaved individuals developed depression. |

^a^ Terms in square brackets identify output provided by *med4way* using the Stata statistical software package (Discacciati et al., 2019). Mathematical notation is also provided. *A* denotes the exposure where *a* = 1 for suicide-bereavement and *a* = 0 for other-bereavement. *M* is the candidate mediator (i.e. depression), where *m* = *1* represents the value of the mediator in the presence of the exposure, and *m* = 0 in its absence. *M*_a_ represents the counterfactual value of the mediator when the exposure *A* is set *A* = *a*. *Y* denotes the outcome (i.e., suicide), where *Y*_am_ is a counterfactual outcome when the exposure *A is* set to *A* = *a* and the mediator *M* is set to *M* = *m.*

Definitions are based on those provided in selected literature (Richiardi et al., 2013), (Vanderweele, 2014b), (Vanderweele, 2016), (Discacciati et al., 2019).

^c^ Definitions are applied to one putative mediator (depression) used in the current study.

Supplemental Figure 1. Log-log plot to assess proportionality of hazards


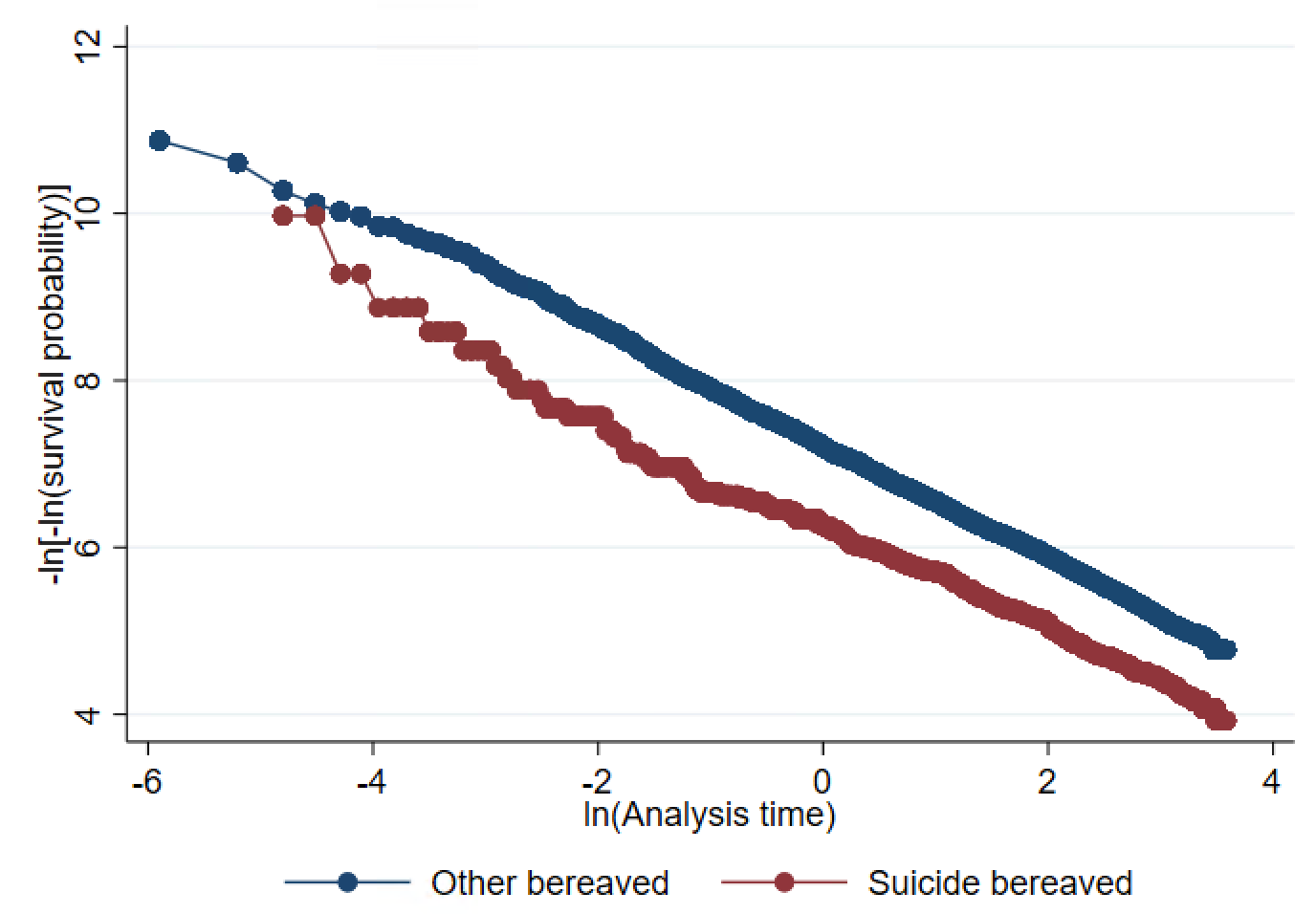
Legend: The plot shows approximately parallel lines for the suicide bereaved (lower trajectory) and other bereaved (upper trajectory) groups, suggesting approximate proportionality of hazards.

Supplemental Table 2. Demographic and clinical features of the analytic sample with complete information and the excluded sample with missing information

|  | **Analytic Sample** | | **Excluded Sample** | | **Test statistic (df), p-value^a^** |
| --- | --- | --- | --- | --- | --- |
| **Characteristic^b^** | **N** | **%** | **N** | **%** |  |
| **Total** | 936,070 | 97.5 | 24,203 | 2.5 | - |
| **Sex** |  |  |  |  | 76.8 (1), <0.001 |
| Male | 306,331 | 32.7 | 7,273 | 30.0 |  |
| Female | 629,739 | 67.3 | 16,930 | 70.0 |  |
| **Age (median, IQR)** | **Median** | **IQR** | **Median** | **IQR** |  |
| Age at bereavement (entry) | 68 | 58-77 | 67 | 59-75 | 9.0, <0.001 |
| Age at exit | 79 | 69-86 | 82 | 75-88 | -53.5, <0.001 |
| **Bereavement year^c^** |  |  |  |  |  |
|  | 1998 | 1990-2007 | 1980 | 1980-1980 | 263.5,<0.001 |
| **Status of deceased partner** | **N** | **%** | **N** | **%** | 7.2x10^3^ (2), <0.001 |
| Current partner | 703,017 | 75.1 | 23,924 | 98.9 |  |
| Ex-partner | 232,842 | 24.9 | 276 | 1.1 |  |
| Missing | 211 | <0.1 | 3 | <0.1 |  |
|  | **Median** | **IQR** | **Median** | **IQR** |  |
| Time since split for ex-partners (years) | 9 | 3-18 | 11 | 0-21 | 0.9, 0.369 |
| **Household income level^d^** | **N** | **%** | **N** | **%** | 0.8 (3), 0.844 |
| 1 (lowest) | 59,698 | 6.4 | 0 | 0.0 |  |
| 2 | 513,096 | 54.8 | <3 | <0.1 |  |
| 3 | 211,601 | 22.6 | 0 | 0.0 |  |
| 4 (highest) | 151,675 | 16.2 | 0 | 0.0 |  |
| Missing | 0 | 0.0 | 24,202 | 100.0 |  |
| **Marital status at bereavement**^e^ |  |  |  |  | 2.8x10^3^ (3), <0.001 |
| Never married | 52,231 | 5.6 | 552 | 2.3 |  |
| Married/registered partnership | 766,764 | 81.9 | 22,714 | 93.9 |  |
| Divorced/dissolved partnership | 103,144 | 11.0 | 428 | 1.8 |  |
| Widowed | 13,931 | 1.5 | 447 | 1.9 |  |
| Missing | 0 | 0.0 | 62 | 0.3 |  |
| **Previous self-harm** | 15,889 | 1.7 | 41 | 0.2 | 337.7 (1) <0.001 |
| **Previous psychiatric disorder** |  |  |  |  |  |
| Any | 58,476 | 6.3 | 666 | 2.8 | 498.7 (1), <0.001 |
| PTSD | 304 | <0.1 | <3 | <0.1 | 4.3 (1), 0.037 |
| Depression | 22,196 | 2.4 | 372 | 1.5 | 71.5 (1), <0.001 |
| Anxiety | 6,563 | 0.7 | 58 | 0.2 | 73.4 (1), <0.001 |
| Substance use | 32,551 | 3.5 | 235 | 1.0 | 449.5 (1), <0.001 |
| SMI | 12,734 | 1.4 | 141 | 0.6 | 107.9 (1), <0.001 |
| **Previous physical disorder** |  |  |  |  |  |
| Any | 150,588 | 16.1 | 973 | 4.0 | 2.6x10^3^ (1), <0.001 |
| Cardiovascular disease | 94,952 | 10.1 | 519 | 2.1 | 1.7x10^3^ (1), <0.001 |
| Hypertension | 31,137 | 3.3 | 121 | 0.5 | 598.5 (1), <0.001 |
| Diabetes mellitus | 22,394 | 2.4 | 227 | 0.9 | 217.0 (1), <0.001 |
| COPD | 26,368 | 2.8 | 150 | 0.6 | 424.1 (1), <0.001 |
| **Exposure** |  |  |  |  | 34.1 (1), <0.001 |
| Suicide bereavement | 913,402 | 97.6 | 23,475 | 97.0 |  |
| Other bereavement | 22,668 | 2.4 | 728 | 3.0 |  |
| **Reason for exit from cohort** |  |  |  |  | 1.2x10^4^ (4), <0.001 |
| Suicide | 3,733 | 0.4 | 189 | 0.8 |  |
| Other death | 493,092 | 52.7 | 20,918 | 86.4 |  |
| Emigration | 3,892 | 0.4 | 93 | 0.4 |  |
| End of follow-up | 393,340 | 42.0 | 1,746 | 7.2 |  |
| Experienced second bereavement | 42,013 | 4.5 | 1,257 | 5.2 |  |

IQR: interquartile range; PTSD: post-traumatic stress disorder; COPD: chronic obstructive pulmonary disorder; SMI: severe mental illness (defined as psychotic disorders, manic episode, bipolar affective disorder, and depression with psychotic symptoms).

Any figures quoted as <3 indicate that cell size was below the threshold for reporting exact figures, as per the Statistics Denmark stipulations on protecting confidentiality. ^a^ P-values to be interpreted in the context of a large sample size, and therefore high probability of statistically significant differences. Compared with the analytic sample, those with missing data were older at cohort entry, more likely to be married and less likely to have a history of self-harm, psychiatric disorder or physical disorder. They were also more likely to be bereaved by suicide and to exit the cohort due to death from other causes or suicide.

^b^ Data are n (%), except age, which is summarised as median (IQR). All values were pre-bereavement unless otherwise specified.

^c^ For 98% of cases in the excluded sample with missing data, their year of bereavement was 1980. This was the year of inception of the Registry of Social Pension and Income, so not all residents would have had income data recorded prior to index bereavement.

^d^ Household income quartiles represent total income within the household divided by the total number of adults living in the household, then categorised into quartiles based on national annual income averages.

^e^ The widowed category represented people who were bereaved by subsequent partner loss before the population registers had started, yet were bereaved whilst in a new partnership, so by default were registered as widowed by a former partner’s death.

Supplemental Table 3. Stepwise adjustments in the associations between a) suicide bereavement and depression, and b) suicide bereavement and substance use

|  | **Depression** | | | **Substance use** | | |
| --- | --- | --- | --- | --- | --- | --- |
|  | **OR** | **95% CI** | **p-value** | **OR** | **95% CI** | **p-value** |
| Unadjusted | 1.43 | 1.34-1.53 | <0.001 | 2.12 | 2.01-2.24 | <0.001 |
| Adjusted for sex | 1.41 | 1.32-1.51 | <0.001 | 2.16 | 2.04-2.28 | <0.001 |
| Adjusted for the above + age | 1.16 | 1.09-1.24 | <0.001 | 0.83† | 0.79-0.88 | <0.001 |
| Adjusted for the above + bereavement year | 1.13 | 1.06-1.21 | <0.001 | 0.81 | 0.77-0.86 | <0.001 |
| Adjusted for the above + marital status | 1.13 | 1.05-1.21 | 0.001 | 0.79 | 0.75-0.84 | <0.001 |
| Adjusted for the above + household income level | 1.14 | 1.07-1.23 | <0.001 | 0.81 | 0.76-0.86 | <0.001 |
| Adjusted for the above + previous SH | 1.14 | 1.07-1.22 | <0.001 | 0.81 | 0.76-0.86 | <0.001 |
| Adjusted for the above + previous psychiatric illness | 1.16 | 1.08-1.25 | <0.001 | 0.83 | 0.78-0.88 | <0.001 |
| Adjusted for the above + previous physical illness | 1.16 | 1.09-1.25 | <0.001 | 0.83 | 0.78-0.88 | <0.001 |

† Older age was associated with a lower probability of substance use (p<0.001); the suicide-bereaved group were significantly younger than the other-bereaved group

Supplemental Table 4. Reason for cohort exit by bereavement status (representing competing risks)

|  | **Suicide Bereavement** | | **Other Bereavement** | |
| --- | --- | --- | --- | --- |
|  | **n** | **%** | **n** | **%** |
| Subsequent partner suicide death^a^ | 101 | 0.5 | 856 | 0.1 |
| Subsequent partner non-suicide death^b^ | 2,294 | 10.1 | 38,762 | 4.2 |
| Death by suicide | 182 | 0.8 | 3,551 | 0.4 |
| Death by non-suicide causes^c^ | 5,886 | 26.0 | 487,206 | 53.3 |
| Emigration^d^ | 424 | 1.9 | 3,468 | 0.4 |
| End of follow-up | 13,781 | 60.8 | 379,559 | 41.6 |

^a^ In this group, 7 people died by suicide after they were censored

^b^ In this group, 89 people died by suicide after they were censored

Note that individuals bereaved by suicide were less likely to die by other causes, and were more likely to migrate out of Denmark and to experience a second partner bereavement (X^2^(4)=8.3x10^3^, p<0.001).

Supplemental Results 1: **Interaction components**

The mediated interaction (INT_med_; the portion of effect due to both mediation by depression and interaction between depression and bereavement) was 0.00 (95% CI: 0.00-0.01, p=0.433), while the reference interaction (INT_ref_; the proportion of effect to interaction between bereavement and depression, but not mediated by depression) was 0.02 (95% CI: -0.02-0.05, p=0.422), ruling out interaction as a source of bias. The PAI (the overall proportion of the effect attributable to interaction between suicide bereavement and depression) was 3.05% (95% CI: -4.47%-10.59%; p=0.427). Results from unadjusted models did not change the interpretation of findings.

Supplemental Table 5: Sensitivity analysis to assess the association between suicide bereavement and suicide risk, accounting for competing risks

|  | **Sub-distribution HR** | **95% CI** |
| --- | --- | --- |
| Unadjusted | 1.99 | 1.71-2.31 |
| Adjusted 1^a^ | 1.59 | 1.36-1.86 |
| Adjusted 2^b^ | 1.54 | 1.32-1.80 |

HR: hazard ratio; CI: confidence interval

^a^ Adjusted for sex, age, bereavement year, marital status and household income level.

^b^ Adjusted for all variables in adjustment 1, plus pre-bereavement history of self-harm, psychiatric disorders, and physical disorders.

Supplemental Table 6. Hazard ratios and 95% confidence intervals for the association between suicide bereavement and suicide, stratified by partner status

|  | **Unadjusted** | | | **Adjusted 1^a^** | | | **Adjusted 2^b^** | | |
| --- | --- | --- | --- | --- | --- | --- | --- | --- | --- |
|  | **HR** | **95% CI** | **P-value for interaction test** | **HR** | **95% CI** | **P-value for interaction test** | **HR** | **95% CI** | **P-value for interaction test** |
| Ex-partner | 1.39 | 1.04-1.84 |  | 1.33 | 1.00-1.77 |  | 1.36 | 1.02-1.81 |  |
| Current partner | 1.91 | 1.60-2.27 | 0.061 | 1.88 | 1.57-2.25 | 0.043 | 1.76 | 1.47-2.10 | 0.130 |

HR: hazard ratio; CI: confidence interval

^a^ Adjusted for sex, age, bereavement year, marital status and household income level.

^b^ Adjusted for all variables in adjustment 1, plus pre-bereavement history of self-harm, psychiatric disorders, and physical disorders.

Supplemental Table 7. Sensitivity analysis to assess the influence of using inpatient admission data only on each pathway of the mediation models

|  |  |  | **Unadjusted** | | **Adjusted 1^a^** | | **Adjusted 2^b^** | |
| --- | --- | --- | --- | --- | --- | --- | --- | --- |
| **Model** | **Group** | **n (%) ^c^** | **OR** | **95% CI** | **OR** | **95% CI** | **OR** | **95% CI** |
| **Depression^d^** |  |  |  |  |  |  |  |  |
| Path A (exposure-mediator) | Other-bereaved | 13,644 (1.5%) | - | - | - | - | - | - |
|  | Suicide-bereaved | 533 (2.4%) | 1.59* | 1.45-1.73 | 1.05 | 0.95-1.14 | 1.06 | 0.97-1.17 |
| Path B (mediator-outcome) | No depression | 3,291 (0.4%) | - | - | - | - | - | - |
|  | Depression | 442 (3.1%) | 8.98* | 8.12-9.93 | 8.53* | 7.70-9.45 | 5.57* | 4.99-6.22 |
| **Substance use^d^** |  | | | | | | | |
| Path A (exposure-mediator) | Other-bereaved | 25,714 (2.8%) | - | - | - | - | - | - |
|  | Suicide-bereaved | 1,330 (5.9%) | 2.15* | 2.03-2.28 | 0.81* | 0.76-0.85 | 0.82* | 0.77-0.88 |
| Path B (mediator-outcome) | No substance use | 3,362 (0.4%) | - | - | - | - | - | - |
|  | Substance use | 371 (1.4%) | 3.75* | 3.36-4.17 | 2.82* | 2.52-3.16 | 1.61* | 1.42-1.82 |

OR: odds ratio; CI: confidence interval

^a^ Adjusted for sex, age, bereavement year, marital status and household income level.

^b^ Adjusted for all variables in adjustment 1, plus pre-bereavement history of self-harm, psychiatric disorders, and physical disorders.

^c^ n is solely for the outcome in each path; thus of those who are suicide-bereaved, 533 (2.4%) have depression and 1,330 (5.9%) have substance use, and of those who are other-bereaved, 13,644 (1.5%) have depression and 25,714 (2.8%) have substance use. Of those who have depression, 442 (3.1%) die by suicide, and of those who do not have depression, 3,291 (0.4%) die by suicide. Similarly, of those who have substance use, 371 (1.4%) die by suicide, and of those who do not have substance use, 3,362 (0.4%) die by suicide.

**^d^**  each mediator modelled separately to assess mediation of the association between suicide bereavement and suicide by depression (Figure 1, Panel A) and substance use (Figure 1, Panel B)* *p*<0.001

Supplemental Table 8. Proportions of sample with multiple bereavements

|  | **Suicide Bereavement** | | **Other Bereavement** | |
| --- | --- | --- | --- | --- |
|  | **n** | **%** | **n** | **%** |
| One subsequent bereavement | 2,109 | 9.3 | 36,212 | 4.0 |
| Suicide death | 89 | 0.4 | 732 | <0.1 |
| Non-suicide death | 2,020 | 8.9 | 35,480 | 3.9 |
| Two or more subsequent bereavements | 286 | 1.3 | 3,410 | 0.4 |
| Suicide death only | 3 | <0.1 | 5 | <0.1 |
| Non-suicide death only | 269 | 1.2 | 3206 | 0.4 |
| Both suicide-death and non-suicide death | 14 | <0.1 | 199 | <0.1 |

Note: individuals were censored at any partner bereavement subsequent to the first (index) partner bereavement. Thus, these figures list outcomes not included in our analysis and are provided to illustrate patterns of repeat exposures.

Supplemental Table 9. Sensitivity analysis for the depression mediation model, excluding those with widowed marital status

|  |  | Adjusted estimate^a^ | | |
| --- | --- | --- | --- | --- |
| **Component** | **Stata output label**^b^ | **RER** | **95% CI** | **p-value** |
| Total effect (TE) | *tereri* | 0.57 | 0.32-0.83 | <0.001 |
| Total effect (TE) Relative Risk Ratio (TE + 1) | *tereria* | 1.57 | 1.32-1.83 | <0.001 |
| **Decomposed into:** |  |  |  |  |
| a) Controlled direct effect (CDE) | *ereri_cde* | 0.55 | 0.29-0.80 | <0.001 |
| b) Reference interaction (INT_ref_) | *ereri_intref* | 0.02 | -0.02-0.06 | 0.359 |
| c) Mediated interaction (INT_med_) | *ereri_intmed* | 0.00 | 0.00-0.01 | 0.374 |
| d) Pure indirect effect (PIE) | *ereri_pie* | 0.01 | 0.00-0.01 | <0.001 |
| **Combinations:** |  |  |  |  |
| Total indirect effect (TIE) | computed | 0.01 | 0.00-0.02 | 0.010 |
| Portion attributable to interaction (PAI) | computed | 0.02 | -0.02-0.06 | 0.360 |
| Portion eliminated (PE) | computed | 0.03 | -0.02-0.07 | 0.213 |
| **Proportions derived:** |  |  |  |  |
| % attributable to CDE | *p_cde* | 95.17 | 87.38-102.96 | <0.001 |
| % attributable to INT_ref_ | *p_intref* | 3.09 | -3.60-9.78 | 0.365 |
| % attributable to INT_med_ | *p_intmed* | 0.45 | -0.55-1.44 | 0.379 |
| % attributable to PIE | *p_pie* | 1.29 | 0.39-2.19 | 0.005 |
| Overall % attributable to mediation by depression  (equivalent to 100*TIE/TE) | *op_m* | 1.74 | 0.24-3.24 | 0.023 |
| Overall % attributable to interaction  (equivalent to 100*PAI/TE) | *op_ati* | 3.53 | -4.12-11.20 | 0.366 |
| % eliminated  (equivalent to 100*PE/TE) | *op_e* | 4.83 | -2.96-12.62 | 0.225 |

RER: relative excess risk; CI: confidence interval; see Supplemental Box 1 for definitions.

^a^ Adjusted for sex, age, bereavement year, marital status, household income level, pre-bereavement history of self-harm, psychiatric disorders, and physical disorders. Note that in a mediation analysis only adjusted estimates are presented because mediation can only be interpreted in the context of adjusting for all measured confounders (see Supplemental Methods 4).

^b^ Where no Stata label is given, this value was computed from other Stata output values.

Note that we only ran this model for depression as there was no evidence to support a mediating effect for substance use.

# Supplemental References

Baadsgaard, M., & Quitzau, J. (2011). Danish registers on personal income and transfer payments. *Scandinavian Journal of Public Health*, *39*(7), 103–105. https://doi.org/10.1177/1403494811405098

Danmarks Statistik. (2022). *C_Type*. Danmarks Statistik. https://www.dst.dk/da/Statistik/dokumentation/Times/cpr-oplysninger/c-type

Discacciati, A., Bellavia, A., Lee, J. J., Mazumdar, M., & Valeri, L. (2019). Med4way: A Stata command to investigate mediating and interactive mechanisms using the four-way effect decomposition. *International Journal of Epidemiology*, *48*(1), 15–20. https://doi.org/10.1093/ije/dyy236

Fine, J. P., & Gray, R. J. (1999). A Proportional Hazards Model for the Subdistribution of a Competing Risk. *Journal of the American Statistical Association*. https://doi.org/10.1080/01621459.1999.10474144

Imai, K., Keele, L., & Tingley, D. (2010). A General Approach to Causal Mediation Analysis. *Psychological Methods*, *15*(4), 309–334. https://doi.org/10.1037/a0020761

Imai, K., Keele, L., & Yamamoto, T. (2010). Identification, Inference and Sensitivity Analysis for Causal Mediation Effects. *ArXiv.Org*, *25*(1). https://doi.org/10.1214/10-STS321

Kposowa, A. J. (2000). Marital status and suicide in the National Longitudinal Mortality Study. *Journal of Epidemiology and Community Health*, *54*(4), 254–261. https://doi.org/10.1136/jech.54.4.254

Richiardi, L., Bellocco, R., & Zugna, D. (2013). Mediation analysis in epidemiology: Methods, interpretation and bias. *International Journal of Epidemiology*, *42*(5). https://doi.org/10.1093/ije/dyt127

VanderWeele, T., & Vansteelandt, S. (2013). Mediation analysis with multiple mediators. *Epidemiologic Methods*, *2*(1), 95–115. https://doi.org/10.1515/em-2012-0010

VanderWeele, T. J. (2011). Causal mediation analysis with survival data. In *Epidemiology*. https://doi.org/10.1097/EDE.0b013e31821db37e

Vanderweele, T. J. (2014a). A unification of mediation and interaction: A 4-way decomposition. *Epidemiology*. https://doi.org/10.1097/EDE.0000000000000121

Vanderweele, T. J. (2014b). A unification of mediation and interaction: A 4-way decomposition. *Epidemiology*, *25*(5), 749–761. https://doi.org/10.1097/EDE.0000000000000121

Vanderweele, T. J. (2016). Commentary: On Causes, Causal Inference, and Potential Outcomes. *International Journal of Epidemiology*, *45*(6), 1809–1816. https://doi.org/10.1093/ije/dyw230
